# TOWARD AN UNDERSTANDING OF IN-HOSPITAL INACTIVITY

**DOI:** 10.1093/geroni/igae098.1794

**Published:** 2024-12-31

**Authors:** Mette Merete Pedersen, Maria Hansen, Thomas Bandholm, Jeanette Kirk

**Affiliations:** Copenhagen University Hospital Hvidovre, Copenhagen, Hovedstaden, Denmark; Physical Medicine & Rehabilitation Research - Copenhagen (PMR-C), Department of Physical and Occupational Therapy, Copenhagen University Hospital Hvidovre, Hvidovre, Hovedstaden, Denmark; Physical Medicine & Rehabilitation Research - Copenhagen (PMR-C), Department of Clinical Research, Copenhagen University Hospital Hvidovre, Hvidovre, Hovedstaden, Denmark; Copenhagen University Hospital Hvidovre, Hvidovre, Hovedstaden, Denmark

## Abstract

Lack of mobility during hospitalization is a common problem in older adults (+65) and is associated with an elevated risk of hospital-associated disability. In a recent study, we have shown that there is unclarity among health professionals on priorities and responsibilities concerning mobility, and barriers to the promotion of mobility are present. Also, in recent years, it has become clear that in-hospital inactivity is rooted in the hospital culture and that multi-dimensional and multi-professional interventions are required to incorporate and implement mobility into clinical practice. However, important factors for such interventions to succeed are patient and caregiver engagement and interaction. Therefore, for future interventions to be successful in routine care it is important to know what characterizes clinical practice and communication around physical activity and what influences current levels of activity. We will present data from clinical studies on older adults (65+) in a geriatric and an orthopedic ward in which we have investigated 1) current and previous levels of physical activity during acute hospitalization; 2) clinical practice about mobility and physical activity - how local culture mediates care routines; and 3) if and how a technological artifact can mediate increased physical activity. Also, we will include the perspective of patients and ward staff on what influences mobility and levels of physical activity among patients hospitalized following hip fractures and for geriatric conditions.

